# Effect of Decitabine (5-aza-2ˈ-deoxycytidine, 5-aza-CdR) in Comparison with Vorinostat (Suberoylanilide Hydroxamic Acid, SAHA) on *DNMT1, DNMT3a and DNMT3b, HDAC 1-3, SOCS 1, SOCS 3, JAK2*, and* STAT3* Gene Expression in Hepatocellular Carcinoma HLE and LCL-PI 11 Cell Lines

**DOI:** 10.31557/APJCP.2021.22.7.2089

**Published:** 2021-07

**Authors:** Masumeh Sanaei, Fraidoon Kavoosi, Mohmmad Pourahmadi

**Affiliations:** 1 *Research Center for Non-Communicable Diseases, Jahrom University of Medical Sciences, Jahrom, Iran. *; 2 *Departments of Anatomical Sciences, Jahrom University of Medical Sciences, Jahrom, Iran. *

**Keywords:** Decitabine, vorinostat, SOCS, JAK/STAT, HCC

## Abstract

**Background:**

Epigenetic alterations play an important role in tumorigenesis. Hypermethylation of CpG islands within the promoter regions of tumor suppressor genes (TSGs) and histone deacetylation lead to the silencing of the genes resulting in cancer induction. The suppressor of cytokine signaling (SOCS) family is an important negative regulator of cytokine signaling and deregulation of this family has been reported in several cancers, the protein of the SOCS family inhibit the cytokine-activated Janus kinase/signal transducers and activators of transcription (JAK/STAT) signaling pathway to modulate cellular responses. Previously, we evaluated the effects of DNA demethylating agents and histone deacetylase inhibitors on hepatocellular carcinoma (HCC). The current study aimed to investigate the effect of decitabine (5-aza-2ˈ-deoxycytidine, 5-aza-CdR) in comparison to vorinostat (suberoylanilide hydroxamic acid, SAHA) on* DNMT1, DNMT3a* and *DNMT3b, HDAC 1-3, SOCS 1, SOCS 3, JAK2*, and* STAT3* gene expression, cell growth inhibition, and apoptosis induction of HCC HLE and LCL-PI 11 cell lines.

**Material and Methods:**

The HLE and LCL-PI 11 cells were treated with 5-aza-CdR and SAHA and then the MTT assay, flow cytometry assay, and quantitative real-time RT-PCR were achieved to determine cell viability, cell apoptosis, and relative gene expression respectively.

**Results:**

The result indicated that both compounds inhibited cell growth, induced apoptosis, and down-regulated *DNMT1, DNMT3a DNMT3b, HDAC 1-3, JAK2*, and *STAT3* and up-regulated *HDAC 1-3, SOCS 1*, and *SOCS* 3 genes expression significantly. The apoptotic effect of SAHA was stronger than that of 5-Aza-CdR.

**Conclusion:**

5-Aza-CdR and SAHA can induce cell growth inhibition and apoptosis induction through the JAK/STAT pathway.

## Introduction

Hepatocellular carcinoma (HCC) is one of the most prevalent and lethal cancers worldwide. The molecular mechanisms and pathways leading to the diseases are extremely complicated including genomic, genetic, and epigenetic changes (Pogribny et al., 2014). There are several evidences that epigenetic alterations play an important role in tumorigenesis, cancer progression, and pathogenesis. These epigenetic changes include DNA methylation and histone modifications which lead to heritable changes in gene expression without alterations in chromatin structure. Hypermethylation of CpG islands within the promoter regions of tumor suppressor genes (TSGs) leads to the silencing of the genes resulting in cancer induction. In HCC, hypermethylation of TSGs involved in cancer induction and progression has been demonstrated (Hoshida et al., 2010). DNA methyltransferases (DNMTs) are the enzymes that are overexpressed in cancer cells. DNMTs transfer a methyl group to one of the four bases that constitute the coding sequence of DNA and catalyze the methylation of CpG islands resulting in DNA methylation. Mammalian DNMTs can be divided into *DNMT1, DNMT3a, DNMT3b*, and *DNMT3l* (Turek-Plewa et al., 2005). 

A significant increase in the mRNA levels of DNMT1, DNMT3a, and DNMT3b has been reported in HCCs (Oh. Et al., 2007). Two DNMT inhibitors (DNMTIs) have been approved by the US Food and Drug Administration (FDA) including decitabine (5-aza-2ˈ-deoxycytidine, 5-aza-CdR) and azacytidine (Vidaza; Celgene) (Gnyszka et al., 2013). In addition to DNMTIs, flavonoids are a class of plant secondary metabolites that have been reported to interfere in the prevention and initiation of cancer by modulating different mechanisms and pathways related to cellular differentiation, proliferation, and apoptosis (Ravishankar et al., 2013). It has been demonstrated that bioflavonoids such as quercetin, genistein, fisetin can inhibit DNMT activity (Nebbioso et al., 2012). Previously, we evaluated the effects of DNA demethylating agent 5-aza-CdR on *DNMT1* gene expression and apoptosis induction and also cell viability in the HCC WCH-17 cell line [Kavoosi et al., 2019; Sanaei et al., 2020; Sanaei et al., 2019). 

As mentioned above, histone modification modulates chromatin structure and affects gene transcription and expression. This modification is a reversible process and result of a balance between the opposing activities of histone deacetylases (HDACs) and histone acetyltransferases (HATs). There are 18 potential human HDACs classified into four classes, including class I (HDAC1, 2, 3, and 8), class II which divided into two subclasses: IIa (HDAC4, 5, 6, 7, and 9) and IIb (HDAC6 and 10), class III which are NAD-dependent protein deacetylases and/or ADP ribosylases and class IV which contains only HDAC11 (Li et al., 2016). By removal of acetyl groups from histones, these enzymes create a chromatin conformation that prevents the transcription of genes that encode several proteins involved in both cancer initiation and cancer progression (Glozak et al., 2007). HDAC1-3 activity increase HCC (Quint et al., 2011; Rikimaru et al., 2007). HDAC inhibitors (HDACIs) can regulate gene expression through the acetylation of histones and non-histone proteins without changing the DNA sequence. Based on chemical structure, these agents can be classified into several groups, including hydroxamic acids [vorinostat, (suberoylanilide hydroxamic acid, SAHA)], aminobenzamides (mocetinostat, entinostat), carboxylic acids (sodium butyrate and valproate), epoxyketones (trapoxins), cyclic peptides (romidepsin and apicidin), and hybrid molecules (West. et al., 2014). The suppressor of cytokine signaling (SOCS) family is an important negative regulator of cytokine signaling and deregulation of this family has been reported in several cancers, the protein of the SOCS family plays a key role in the negative regulation of cytokine signal transduction. These proteins inhibit the cytokine-activated Janus kinase/signal transducers and activators of transcription (JAK/STAT) signaling pathways to modulate cellular responses. The SOCS family consists of eight members, including SOCS-1 to SOCS-7 and cytokine-inducible SH2 protein. SOCS1 and 3 appear to have tumor suppressor activity in HCC cells (Kim et al., 2015; Puhr et al., 2009). Hypermethylation and deacetylation of the SOCS family have been shown in numerous solid cancers (Kim. Et al., 2015). Our previous finding indicated that histone deacetylase inhibitors valproic acid (VPA) and trichostatin A (TSA) can induce apoptosis in HCC (Kavoosi et al., 2018; Sanaei et al., 2018; Sanaei et al., 2017; Sanaei et al., 2019). The aim of the current study was to investigate the effect of 5-aza-CdR in comparison to SAHA on *DNMT1, DNMT3a* and *DNMT3b, HDAC 1-3, SOCS 1, SOCS 3, JAK2, *and *STAT3* gene expression, cell growth inhibition, and apoptosis induction of HCC HLE and LCL-PI 11 cell lines.

## Materials and Methods

*Materials*

The human hepatocellular carcinoma HLE and LCL-PI 11 cell lines were purchased from the National Cell Bank of Iran Pasteur Institute. 5-aza-CdR, SAHA, 3-(4,5-dimethyl-2-thiazyl)-2,5-diphenyl-2H-tetrazolium bromide (MTT), and Dulbecco’s modified Eagle medium (DMEM) were supplied by Sigma–Aldrich (Sigma–Aldrich, Louis, MI, USA). The Annexin V and propidium iodide (PI) apoptosis kit was purchased from Life Technologies. Dimethyl sulfoxide (DMSO) was purchased from Merck Co. (Darmstadt, Germany). Total RNA extraction kit (TRIZOL reagent) and real-time polymerase chain reaction (PCR) kits (qPCR MasterMix Plus for SYBR Green I dNTP) were obtained from Applied Biosystems Inc. (Foster, CA, USA). This work was approved by the Ethics Committee of Jahrom University of Medical science with a code number of IR.JUMS.REC.1396.154.

*Cell culture*

Both cell lines were maintained in DMEM supplemented with 10% fetal bovine serum (FBS), 1% nonessential amino acids, 1% antibiotics (penicillin/streptomycin), and grown in a humidified incubator at 37°C containing 5% CO2. SAHA and 5-Aza-CdR were dissolved at a concentration of 100 μM in DMSO to prepare a stock solution and all of the other test concentrations were provided by dilution of this solution. The final DMSO concentration did not exceed 0.1% and all control groups were administered 0.1% DMSO concentration. 

*Cell viability assay*

The effect of 5-Aza-CdR and SAHA on the HLE and LCL-PI 11 cell viability was measured by MTT assay. First, the HLE and LCL-PI 11 cells were cultured with a culture medium. After enough confluency (more than 80%), 4 ×105 cells per well were transferred into 96-well plates and allowed to adhere overnight. After 24 hours of cell seeding, the cells were treated with medium containing different doses of 5-Aza-CdR (1, 2.5, 5, 10, and 20 μM) and SAHA (1, 2.5, 5, 10, and 20 μM) except control groups for different periods, an equal volume of solvent, DMSO, was added to control experiments. After exposure to the various concentrations of the compounds for 24 and 48 h, the cells were trypsinized and the viable cell population was determined using the MTT assay. 

*Flow cytometric analysis of apoptosis*

To detection apoptotic cells, the apoptotic cells were assessed using the Annexin V-FITC/PI detection kit. In this regard, the HLE and LCL-PI 11 cells were cultured in 24-well plates at a density of 4 × 10^5^ cells/well and incubated for 24h before treatment with a medium containing SAHA and 5-Aza-CdR. After 24h, the cells treated with 5-Aza-CdR and SAHA, based on IC_50_ values indicated in table 1, for 24 and 48h. After treatment times, all the adherent cells were harvested with trypsin- EDTA washed with cold PBS, and resuspended in Binding buffer (1×). Annexin-V-(FITC) and PI were used for staining according to the protocol. Finally, the apoptotic cells were counted by the FACScanTM flow cytometer (Becton Dickinson, Heidelberg, Germany).

*Determination of gene expression by quantitative real-time RT-PCR*

To determine *DNMT1, DNMT3a, DNMT3b, HDAC 1-3, SOCS 1, SOCS 3, JAK2*, and *STAT3* genes expression in HLE and LCL-PI 11 cells, the cells were treated with 5-Aza-CdR and SAHA, based on IC_50_ values indicated in table 1, for 24 and 48 h. After exposure to the compounds, the total RNA of the control and treated cells was extracted using the RNeasy Mini Kit (Qiagen, Valencia, CA, USA) according to the manufacturer’s protocol and then pretreated with RNase-free DNase (Qiagen) to remove the genomic DNA prior to cDNA synthesis. The RNA concentration was determined using a Biophotometer (Eppendorf, Hamburg, Germany). Total RNA (100 ng) was reverse transcribed to cDNA using the RevertAidTM First Strand cDNA Synthesis Kit (Fermentas, Thermo Fisher Scientific, Waltham, MA, USA) according to the manufacturer’s instructions. Real-time RT-PCR was performed with the Maxima SYBR Green ROX qPCR Master Mix Kit (Fermentas) as described previously (Sanaei et al., 2018). The primer sequences were obtained from our previous and other published articles (Sanaei et al., 2018; Nemati et al., 2018, Sutherland et al., 2004; Zhou et al., 2014; Wang et al., 2017) which their sequences are shown in Table 2. GAPDH was used as an endogenous control.

*Statistical analysis*

The database was set up with the SPSS 16.0 software package (SPSS Inc., Chicago, Illinois, USA) and Graph Pad Prism 8.0 for data analysis. Results are expressed as mean ±standard deviation (SD) for n=3 independent experiments. Statistical comparisons between groups were performed with ANOVA (oneway ANOVA) and the Turkey test. A significant difference was considered as P < 0.05.

## Results

*In vitro effects of 5-Aza-CdR and SAHA on the HLE and LCL-PI 11 cells growth*

**Figure 1:**
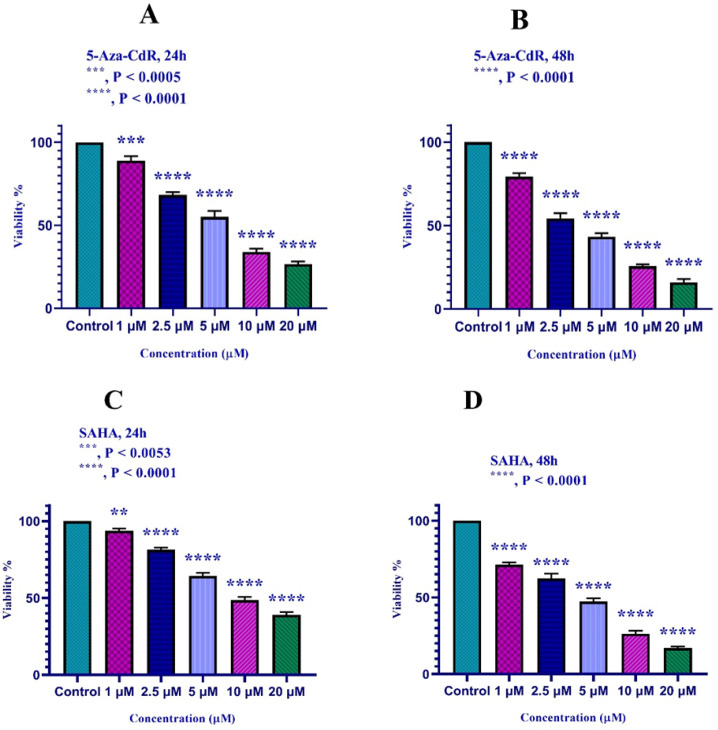
In vitro Effects of 5-Aza-CdR and SAHA on HLE (part A and B) and LCL-PI 11 (part C and D) cells viability. Values are means of three experiments in triplicate. Asterisks (*) demonstrate significant differences between treated and untreated control groups. Results are expressed as mean ±standard deviation (SD) for n=3 independent experiments. Part A: ***, P < 0.0005; ****, P < 0.0001; Part B: ****, P < 0.0001; Part C: ***, P < 0.0053, ****, P < 0.0001; Part D: ****, P < 0.0001

The HLE and LCL-PI 11 cells were treated with different doses of 5-Aza-CdR and SAHA vs control groups for 24 and 48h. As mentioned above, the inhibitory effects of the compounds were evaluated by MTT assay. The results indicated that both compounds can inhibit HLE and LCL-PI 11 cell growth significantly versus control groups. As shown in figure 1, 5-Aza-CdR and SAHA significantly inhibited cell growth with all mentioned doses (p < 0.001). IC_50_ values for 5-Aza-CdR and SAHA are indicated in Table 1.

*Effects of 5-Aza-CdR and SAHA on the HLE and LCL-PI 11 cells apoptosis*

**Figure 2:**
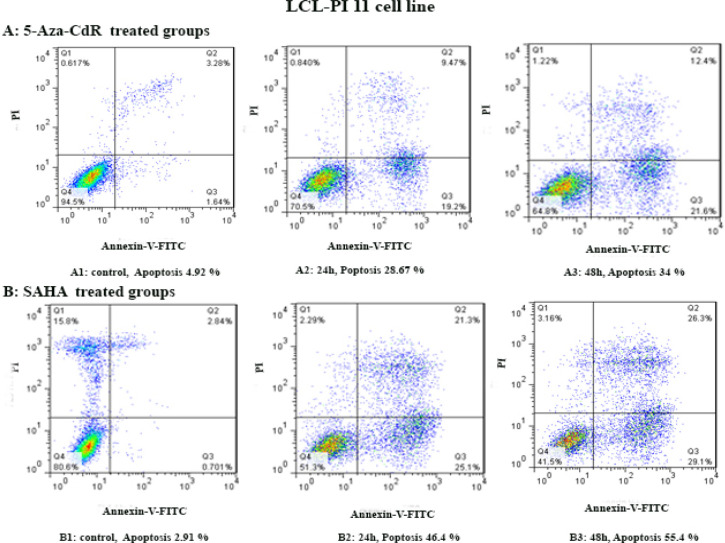
The Apoptotic Effect of 5-Aza-CdR and SAHA on LCL-PI 11 Cell Line. Results were obtained from three independent experiments. Quadrant (Q) 2 and 3, late and primary apoptosis respectively, were calculated in this graph

**Figure 3:**
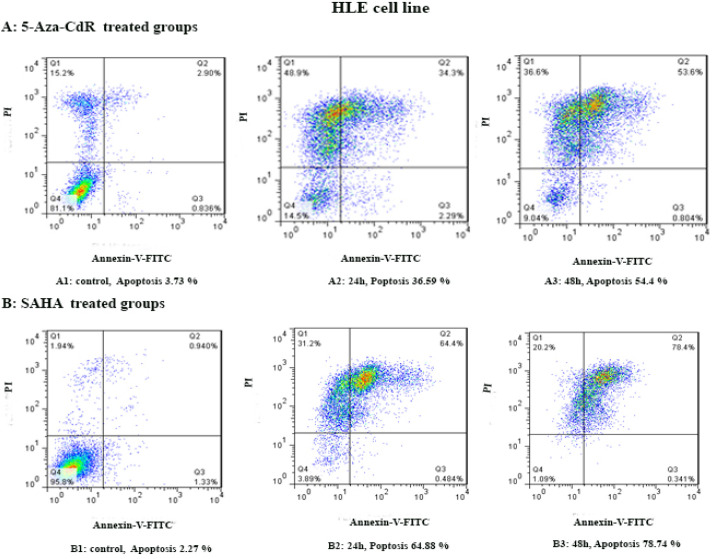
The Apoptotic Effect of 5-Aza-CdR and SAHA on the HLE Cell Line. Results were obtained from three independent experiments. Quadrant (Q) 2 and 3, late and primary apoptosis respectively, were calculated in this graph

**Figure 4:**
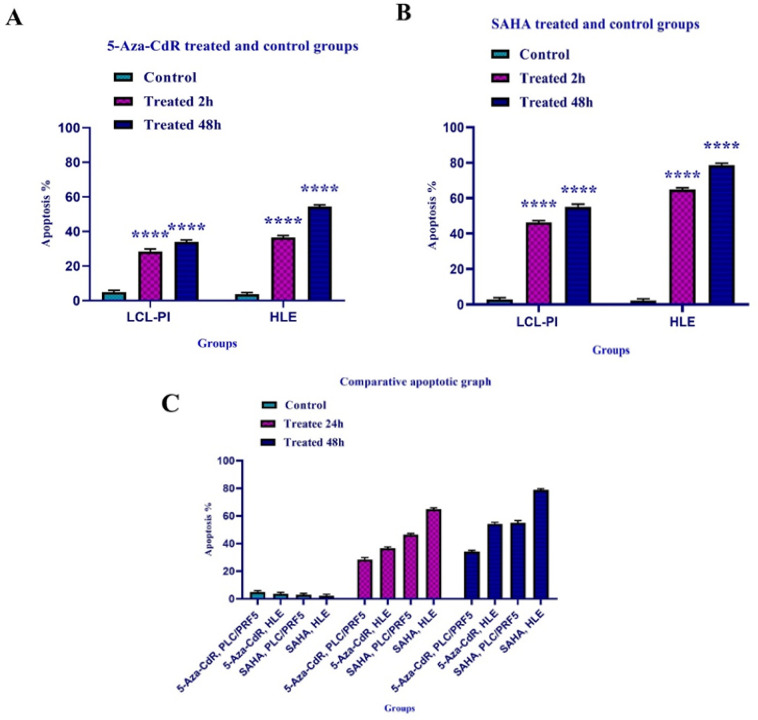
The Apoptotic Effect of 5-Aza-CdR (part A) and SAHA (part B) on HLE and LCL-PI 11 Cells versus Control Groups. The effect of 5-Aza-CdR in comparison to SAHA on HLE and LCL-PI 11 cells has been shown in part C. Results were obtained from three independent experiments and were expressed as mean ± standard error of the mean. The results of the statistical analysis indicate significant differences between treated and untreated cells. ****, P<0.0001

By flow cytometry assay, the apoptotic cells were determined. In this regard, the cells were treated with 5-Aza-CdR and SAHA for 24 and 48 h except for the control groups. Both compounds induced apoptosis significantly as indicated in Figures 2 and 3. The percentage of apoptotic cells is indicated in table 3. Relative analysis indicated that SAHA induced apoptosis more significantly than 5-Aza-CdR (P<0.0001), Figure 4. Maximal apoptosis was seen in the group which received SAHA for 48h.

*Effects of 5-Aza-CdR and SAHA on gene expression*

**Figure 5:**
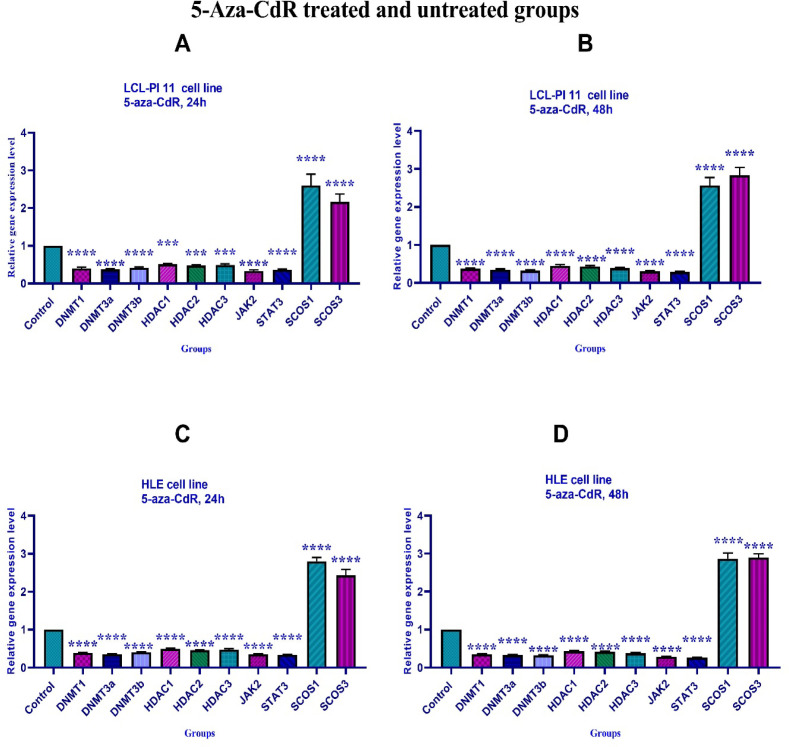
The Relative Expression Level of *DNMT1, DNMT3a, DNMT3b, HDAC 1-3, SOCS 1, SOCS 3, JAK2*, and *STAT3* Genes in the HLE and LCL-PI 11 Cell Lines Treated with 5-Aza-CdR. A significant difference was seen between treated and untreated control groups. Asterisks (*) indicate significant differences between the treated and untreated control groups. Results are expressed as mean±standard deviation (SD) for n=3 independent experiments. Part A: ***, P<0.0008, ****, P<0.0001; Part B: ****, P<0.0001; Part C: ****, P<0.0001; Part D: ****, P<0.0001

**Figure 6:**
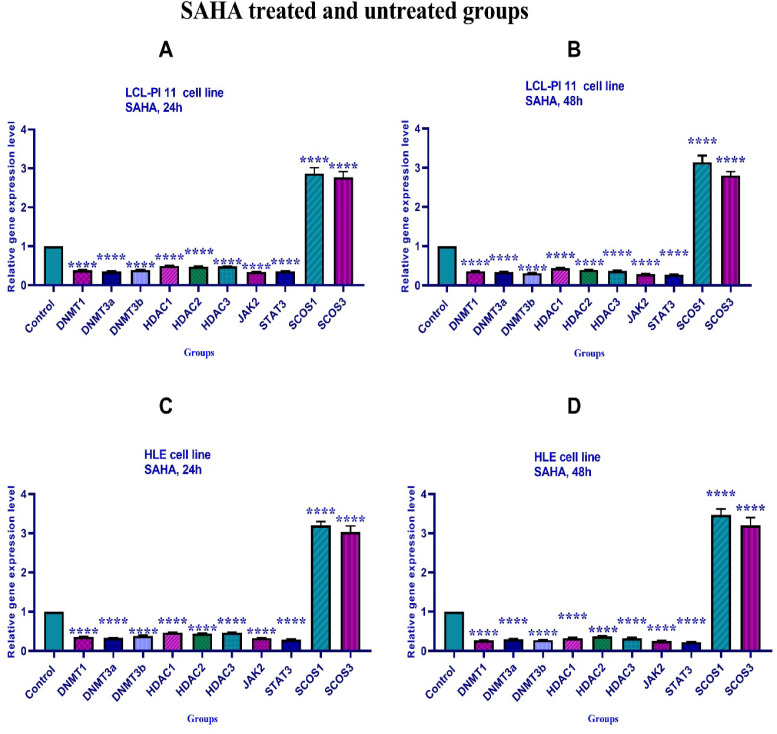
The Relative Expression Level of *DNMT1, DNMT3a, DNMT3b, HDAC 1-3, SOCS 1, SOCS 3, JAK2,* and *STAT3* Genes in the HLE and LCL-PI 11 Cell Lines Treated with SAHA. A significant difference was seen between treated and untreated control groups. Asterisks (*) indicate significant differences between the treated and untreated control groups. Results are expressed as mean±standard deviation (SD) for n=3 independent experiments. Part A: ****, P<0.0001; Part B: ****, P<0.0001; Part C: ****, P<0.0001; Part D: ****, P<0.0001

To determine the effect of 5-Aza-CdR and SAHA on *DNMT1, DNMT3a, DNMT3b, HDAC 1-3, SOCS 1, SOCS 3, JAK2*, and *STAT3* genes expression, real-time RT-PCR was carried out. The results indicated that both compounds significantly down-regulated *DNMT1, DNMT3a, DNMT3b, HDAC 1-3, JAK2*, and *STAT3* and up-regulated *SOCS 1*, and *SOCS 3* gene expression at different periods (24 and 48 h), Figures 5 and 6. 

**Table 1:** IC_50_ Values

Drug	Duration/h	IC_50_ value/ μM	LogIC_50_	R squared
5-Aza-CdR	24	2.62	0.4193	0.9765
5-Aza-CdR	48	1.4	0.1464	0.9478
SAHA	24	4.19	0.6228	0.989
SAHA	48	3.37	0.5278	0.9064

**Table 2:** Real-Time PCR Primers Sequences Used in the Current Study

Primer name	Primer sequences (5’ to 3’)	Product length	Reference
DNMT1 Forward	GAG GAA GCT GCT AAG GAC TAG TTC	206	23
DNMT1 Reverse	ACT CCA CAA TTT GAT CAC TAA ATC		23
DNMT3a Forward	GGA GGC TGA GAA GAA AGC CAA GGT	370	23
DNMT3a Reverse	TTT GCC GTC TCC GAA CCA CAT GAC		23
DNMT3b Forward	TAC ACA GAC GTG TCC AAC ATG GGC	195	23
DNMT3b Reverse	GGA TGC CTT CAG GAA TCA CAC CTC		23
HDAC 1 Forward	AAC TGG GGA CCT ACGG	230	24
HDAC 1 Reverse	ACT TGG CGT GTC CTT		24
HDAC 2 Forward	GTT GCT CGA TGT TGG AC	212	24
HDAC 2 Reverse	CCA GGT GCA TGA GGTA		24
HDAC 3 Forward	TAGGGATGAGATACAGACAAGG	103	25
HDAC 3 Reverse	GAAGCAGGGAAGAAATAAGG		25
SOCS-1 Forward	AGAC CCCTTCTCACCTCTTG	251	26
SOCS-1 Reverse	CTGCACAGCA GAAAATAAAGC		26
SOCS-3 Forward	TCCCCCCAGAAGAGCCTATTAC	107	26
SOCS-3 Reverse	TCCG ACAGAGATGCTGAAGAGTG		26
JAK2 Forward	AGCCTATCGGCATGGAATATCT	163	27
JAK2 Reverse	TAACACTGCCATCCCAAGACA		27
STAT3 Forward	ACCAGCAGTATAGCCGCTTC	124	27
STAT3 Reverse	GCCACAATCCGGGCAATCT		27
GAPDH Forward	AGCCACATCGCTCAGACAC	66	28
GAPDH Reverse	GCCCAATACGACCAAATCC		28

**Table 3:** The Percentage of Apoptotic Cells Treated with 5-Aza-CdR and SAHA for 24 and 48 h

Drug	Cell line	Duration (h)	Apoptosis (%)	P-value
5-Aza-CdR	LCL-PI 11	24	28.67	0.001
5-Aza-CdR	LCL-PI 11	48	34	0.001
5-Aza-CdR	HLE	24	36.59	0.001
5-Aza-CdR	HLE	48	54.4	0.001
SAHA	LCL-PI 11	24	46.4	0.001
SAHA	LCL-PI 11	48	55.4	0.001
SAHA	HLE	24	64.88	0.001
SAHA	HLE	48	78.74	0.001

## Discussion

Acetylation of lysine is regulated by the opposing activity of two groups of enzymes including HDACs and HATs. The HATs catalyze the transfer of an acetyl group to the ε-amino group of lysine thereby neutralize the lysine’s positive charge by which weak the interactions between histones and DNA and facilitates transcription. There are two major groups of HATs including type-A and Type-B (Bannister et al., 2011). A well-known characteristic of cancer is the deregulation of posttranslational histone modifications in particular histone acetylation. Several studies have demonstrated that a histone deacetylation is a common event in human cancer (Ropero et al., 2007). In addition to histone deacetylation, aberrant expression of DNMTs leads to DNA methylation which is closely associated with tumorigenesis (Jin et al., 2013). Several compounds such as butyric acid, SAHA, and TSA can inhibit HDAC activity thereby inhibit the cell growth of transformed cells and induce apoptosis (Marks et al., 2000). Previously, we reported the effect of zebularine on class I HDACs (HDACs 1, 2, 3) and class II HDACs (*HDACs 4, 5, 6*) gene expression in colon cancer LS 180 and LS 174T cell lines (Sanaei et al., 2020; Sanaei et al., 2020).

DNA methyltransferase inhibitors such as 5-aza-CR and vidaza have recently been approved as an anticancer drugs. The 5-aza-CR is the most widely used DNMTs inhibitor characterized 25 years ago, this compound is a cytidine analog that inhibits DNA methyltransferases (Stresemann et al., 2006). In the present study, we report that 5-Aza-CdR and SAHA significantly down-regulated *DNMT1, DNMT3a, DNMT3b* and *HDAC 1-3, JAK2, *and *STAT3* and up-regulated *SOCS 1*, and *SOCS 3* gene expression at different periods (24 and 48 h) in HLE and LCL-PI 11 cell lines. In line with our report, the inhibitory and apoptotic effect of SAHA has been reported on HCC HepG2 (Wang et al., 2013), cervix cancer, colon, and rectum carcinoma (Anantharaju et al., 2017). As we reported, SAHA could induce apoptosis by down-regulation of DNMTs. Other studies have been reported that SAHA induces the expression of apoptosis signal-regulating kinase 1 (ASK1), which induces apoptosis via death receptor and intracellular apoptotic pathways. Furthermore, this compound activates the expression of pro-apoptotic proteins, including Bak and Bax, and inhibits the expression of anti-apoptosis proteins, including Bcl-xL and Bcl-2 resulting in apoptotic induction in tumor cells. Another pathway is the induction of transcription of CDK inhibitor p21/wafl leads to the reduction of cell proliferation and induction of apoptosis (Tan et al., 2006). SAHA can arrest tumor cells in the G0/G1 phase and induce apoptosis which may be related to the increased expression of *p21/waf1*. It can also induce apoptosis by influencing the expression of* p27* and *p53 *(Hirata et al., 2011; Grimes et al., 2011). Several studies have demonstrated that SAHA can activate caspase-3 to promote apoptosis by activation of TRAIL-DISC in HCC (Liu et al., 2018).

Apoptosis induction of HCC cells by DNMTs down-regulation was one of the results of our work. Inconsistent with our finding, it has been shown that 5-aza-CdR reactivates TSGs through down-regulation of DNMT1, DNMT3a, and 3b resulting in rapid demethylation (Ghoshal et al., 2008). Similarly, it has been indicated that 5-Aza-CdR inhibits the activity of DNMT3a and DNMT3b in gastric cancer cell lines SGC7901 and BGC823 thereby up-regulate tumor suppressor gene RASSF1A. The effect of 5-Aza-CdR co-treatment with sodium butyrate on DNMT3a and DNMT3b inhibition is significantly higher than that of 5-AzaCdR alone (Shen et al., 2008). In mouse hippocampus-derived neuronal HT22 cell line, 5-Aza-CdR down-regulates the expression of mRNA and protein DNMT1 and 3a (but no DNMT 3b) (Yang et al., 2017). In myelodysplastic syndrome (MDS), 5-Aza-CdR can re-activate the expression of the* p15INK4B* gene in MUTZ-1 cells by demethylation of the *p15INK4B* gene through inhibition of *DNMT3a* gene expression which is a mechanism of 5-Aza-CdR in the treatments of MDS (Tong et al., 2004). It has been shown that SAHA inhibits cell growth and induces apoptosis in pancreatic cancer by inhibiting HDAC1, 3, and 4 (Kumagai et al., 2007). In uterine cancer, it has been reported that SAHA efficiently suppresses MES-SA uterine sarcoma cell growth by down-regulation of HDACs class I (HDAC2 and 3) and class II (HDAC7). 

Our findings indicated that SAHA down-regulated HDAC 1-3 significantly. Similarly, our previous work demonstrated that histone deacetylase inhibitor trichostatin A can down-regulate class I HDACs (HDACs 1, 2, 3) and class II HDACs (*HDACs 4, 5, 6*) gene expression in colon cancer LS 180 and LS 174T cell lines (Sanaei et al., 2020; Sanaei et al., 2020). In line with our report, it has been shown that SAHA can down-regulate HDACs class I (HDAC2 and 3) and class II (HDAC7) resulting in p21WAF1 upregulation and apoptosis induction in uterine cancer (Hrzenjak et al., 2010). 

Similar to our result, other researchers have indicated that histone deacetylase inhibitor TSA suppresses the growth of colorectal cancer cells, and induces apoptosis through SOCS1 and SOCS3 upregulation and JAK2/STAT3 signaling inhibition (Xiong et al., 2012). Furthermore, in vitro studies have shown that 5-Aza and TSA can reactivate the *SOCS-3* gene in acute myeloid leukemia (AML) cells (Johan et al., 2015). Inconsistently, our previous work indicated that valproic acid and zebularine can up-regulate *SOCS-1* and *SOCS-3 *gene expression leads to cell growth inhibition in the colon carcinoma SW48 cell line (Sanaei et al., 2020). In summary 5-Aza-CdR and SAHA can induce apoptosis through the JAK/STAT pathway. We didn’t evaluate the protein assessment of the mentioned genes. Therefore, the evaluation of these proteins is recommended.

In conclusion, 5-Aza-CdR and SAHA can epigenetically induce apoptosis through up-regulation of *SOCS 1* and *SOCS 3* gene expression and *JAK2/STAT3* signaling inhibition in HLE and LCL-PI 11 cell lines. 

## Author Contribution Statement

All authors generated the ideas and contributed to the writing of the manuscript, acquisition of data, analysis, interpretation of data, critical revision of the manuscript for important intellectual content, statistical analysis, and technical or material support. All authors approved the final revision.
